# The relative cost-effectiveness of atraumatic needles compared to conventional needles in diagnostic lumbar punctures

**DOI:** 10.1186/s12962-025-00612-0

**Published:** 2026-03-06

**Authors:** James Evans, Julia Lowin, Pippa Anderson

**Affiliations:** 1 The Surgical Materials Testing Laboratory (SMTL), Princess of Wales Hospital, Bridgend, CF31 1RQ UK; 2 Swansea Centre for Health Economics (SCHE), Swansea University, Singleton Park, Swansea, SA2 8PP UK

**Keywords:** Lumbar puncture, Atraumatic needles, PDPH, Cost, Economic model, Resource-use

## Abstract

**Introduction:**

Clinical evidence indicates that atraumatic needles (ATNs) versus conventional needles (CNs) reduce diagnostic lumbar puncture (DLP) complications. Despite this, the use of CNs in DLP remains widespread. This analysis estimates the cost-effectiveness of ATNs versus CNs in DLP.

**Methods:**

We constructed a model mapping DLP patient pathways and complications (limited to PDPH events and PDPH-related hospitalisations/epidural blood patches (EBP)). Model development was carried out in consultation with local clinical experts. Published data informed clinical data inputs (DLP characteristics and likelihood of PDPH) and resource estimates. Costs of PDPH management were estimated from UK NHS Reference Costs. Costs of LP were limited to needle costs. Model outputs included total PDPH, total costs, cost per PDPH avoided and numbers need to treat (NNT) to avoid one case of PDPH. Extensive one-way sensitivity analyses were conducted.

**Results:**

Based on 100 patients undergoing DLP with CN (ATN), we estimated 31 (12) cases of PDPH with 7 (3) patients requiring EBP with total costs estimated at £9,469 (£4,257) i.e. 19 fewer cases of PDPH with ATN at a cost saving of £5,212. NNT to avoid one case of PDPH (hospitalised PDPH) was estimated at 5 (13). Clinical benefits and cost savings were robust to plausible input changes.

**Discussion and conclusion:**

Our model findings support an economic case for use of ATN in preference to CN in DLP, with improved outcomes achieved at a cost saving. Local data collection is recommended but is not expected to change the model findings.

## Key points

We constructed a simple model in collaboration with local clinicians to look at the clinical and cost implications of needle choice in diagnostic lumbar puncture procedures. We found that use of atraumatic needles in preference to currently used conventional needles leads to reduced hospitalisations and costs, alongside improved patient outcomes. We provide an economic argument for choosing atraumatic needles in preference to conventional needles in diagnostic lumbar puncture which we hope will complement already established clinical arguments for their use and uptake in DLP and better support evidence-based commissioning.

## Introduction

Diagnostic lumbar puncture (DLP) involves the insertion of a needle into the spinal canal of a patient to obtain cerebrospinal fluid (CSF). Conventional needles (CN), cutting needles, cut through the dura whilst atraumatic needles (ATN), pencil-point needles, are thought to push through and separate dural fibres. The type of needle influences the likelihood of procedure-related complications.

Clinical evidence is consistent in showing that the use of ATNs for DLP reduces the incidence of potential complications [1, 2]. These complications can range from mild headaches and hearing disturbances to debilitating post-dural puncture headache (PDPH). PDPH occurs as a result of a sustained leak of CSF from a dural tear, within five days of lumbar puncture (LP) and potentially incurs substantial healthcare resource use (HCRU) [2, 3]. If conservative management (bedrest, analgesics or caffeine) does not resolve PDPH, hospitalisation or an epidural blood patch (EBP) procedure may be needed. A recent systematic review, including 110 trials conducted across all LP indications (myelopathy, DLP and anaesthesia), compared outcomes with ATNs and CNs and found that the use of ATNs significantly reduced the risk of subsequent PDPH following DLP procedures (relative risk 0·38, 95% CI 0·26–0·55, *p* < 0·0001) [1].

Despite consistent use of ATNs in anaesthetic LPs and strong clinical evidence for use of ATNs in DLP [1, 2], CNs remain in common use for DLP [4, 5]. It is not clear why this is the case. We carried out a review of published literature and constructed an economic model to help quantify the potential economic implications of needle choice. The work was conducted from the perspective of the UK NHS, with the objective of exploring the economic impact of substituting ATNs in DLP.

## Methods

### Objective

Our objective was to estimate the relative cost-effectiveness of ATNs compared to CNs in DLP.

### Patient population

The population comprised patients undergoing a DLP procedure and at risk of PDPH. The patient characteristics reflected those reported by Nath and colleagues in their recent systematic review (mean age 40 years, 60% female, less than 5% under 18 years of age) [1].

### Perspective and timeframe

The analysis was conducted from a UK NHS and Welsh Local Health Board (LHB) perspective with costs estimated in 2022 UK£. Analysis was limited to direct medical costs. The timeframe of the analysis was set to 14 days, consistent with the timeframe for spontaneous resolution of PDPH [3]. Discounting was not included due to the timeframe.

### Comparators

We compared use of ATNs for DLP against the default standard practice, CNs. All other procedure-related factors were assumed to be the same across the two comparator arms.

### Derivation of the economic model

An economic model was constructed to combine available clinical and economic data. Systematic reviews of clinical and economic literature were conducted with search terms refined in collaboration with local clinicians. The objective of the reviews was to identify (1) robust clinical evidence on which to base estimates of relative effectiveness and (2) previous economic models on which we might base the model design and analytical approach. The clinical and economic terms are included as a technical appendix (Supplementary Materials). Two recent meta-analyses were identified in the clinical search [1, 6]. The meta-analysis conducted by Nath and colleagues [1] was judged most relevant to our decision problem, based on the numbers and locations of included studies, and was used to inform estimates of clinical effectiveness. Four economic studies assessing the cost-effectiveness of different needle choices in DLP were retrieved [7–10] The decision tree reported by Tung and colleagues [8] formed the basis for the initial mapping of our patient pathways. Clinical experts were consulted to confirm current local practice in DLP and PDPH management and validate core model assumptions. In addition, the model and outcomes were comprehensively reviewed by clinical staff and other NHS professionals to ensure our approach was consistent with routine practice and information needs.

### Model structure

**Fig. 1:**
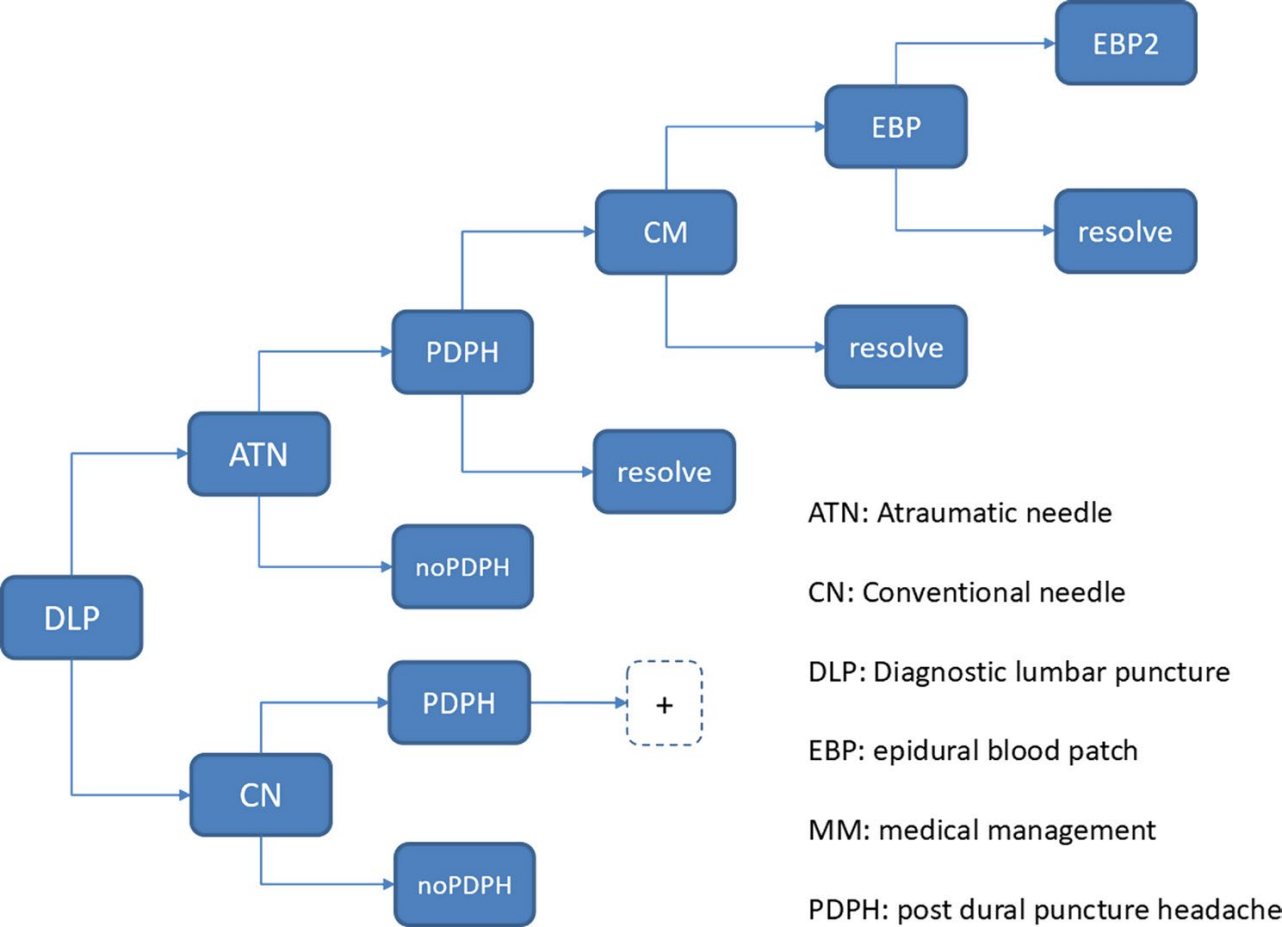
Model schematic

We constructed a decision tree model in Microsoft Excel, based on the model structure reported by Tung and colleagues [8] The model as reported provided a simple, validated and appropriate framework (with minor adaptations) for combining the available clinical data with relevant local costs that was in line with both our decision problem (quantifying the expected cost of a shift from CN to ATN in DLP) and the available evidence (a statistically significant difference in expected rates of PDPH through use of CN or ATN). The model structure is reported in Fig. 1.

### Patient pathway

The pathway for patients undergoing DLP was developed in collaboration with local clinicians. DLP patients could either develop PDPH or remain PDPH-free. PDPH could develop during the initial hospital episode or after discharge. If PDPH developed, management followed the clinical guidelines of the Obstetric Anaesthetists’ Association (OAA) [11, 12] and was validated as in line with local practice.

Modelled management options included no medical management (no NHS cost of treatment); medical management (MM, proxied here by hospital-based management) or, given eligibility and following 24 h of MM, application of an epidural blood patch (EBP). Patients who were not treated successfully with one EBP went on to have a second EBP (the success rate for a second EBP was assumed to be 100%). In line with UK practice, no further treatment options were modelled.

### Clinical inputs

The probability of PDPH and the subsequent probability of medical or surgical management was estimated from the literature.^1^ Baseline rates of PDPH following DLP with CN and the relative risk (RR) of PDPH through use of ATN were estimated from DLP population data [1]. The likelihood of medical and surgical management given PDPH was estimated from LP-level population data (including data from diagnostic, anaesthetic and myelographic populations) under the assumption that management of PDPH would be consistent across the patient populations. The probability of EBP was reported directly and we took the parameter ‘need for IV or controlled analgesia’ as a proxy for hospital readmission/stay (i.e. medical management in our model). The model applied these data to the DLP-specific probability of PDPH to estimate population-specific rates of MM and EBP (in line with our patient pathway). Clinical inputs are reported in Table 1.

**Table 1:** Clinical inputs

Parameter	Reported data	CN input	RR of event	ATN input
p PDPH	0.307 (DLP/CN)	0.307	0.380 (DLP)	0.117
p MM	0.028 (of 0.069^a^)	0.399	NA	0.399
p EBP	0.017 (of 0.028^a^)	0.601	NA	0.601
p second EBP	0.164^b^	0.164	NA	0.164
p DLP failure	0.124 (all CN)	0.124	1.000 (all LP)^c^	0.124

^a^Probability of MM and EBP estimated based on LP-related PDPH events (CN and ATN) (Nath et al. 2018 [1]); ^b^Based on the probability of a second EBP in the UK studies (Russell et al. 2019 [13]); ^c^No significant difference in failure rate between ATN and CN (Nath et al. 2018 [1])

Abbreviations: PDPH: post dural puncture headache; MM: medical management; EBP: epidural blood patch; LP: lumbar punctures (all types); DLP: diagnostic lumbar puncture; CN: conventional needle; ATN: atraumatic needle; NA: not applied (no data specific to DLP); RR: relative risk

### Resource and cost inputs

Resource and costs were estimated based on use of needles and the expected probability of PDPH. The costs associated with the development and management of PDPH were limited to costs associated with hospital-based MM and application of therapeutic EBP. Patient costs were not included. There were very limited data available to estimate PDPH-specific management costs. MM was costed according to the tariff code ‘Headache, Migraine or Cerebrospinal Fluid Leak’ based on reported returns for non-elective inpatient stay (long stay and short stay) (NEL and NES) [13]. We assumed a patient cohort with only moderate comorbidities (in line with the patient population included in the underlying meta-analysis [1]. We therefore included codes with complications and comorbidities (cc) of 10 or less. Costs associated with potential but rare events such as intracranial haemorrhage were not included in our analysis. EBP was costed according to the same tariff ‘Headache, Migraine or Cerebrospinal Fluid Leak’ but based on codes with cc of 11 or more and adjusted to day case costing (in line with feedback from the clinicians consulted for this study) [13]. Management prior to EBP (in or out of hospital) was not costed separately in our model. MM and EBP costs were estimated based on a weighted average of the reported cases. Diagnosis costs were assumed to be incorporated within the finished consultant episode (FCE) codes for MM and EBP admissions and were not incorporated separately for non-hospital-managed patients. The costs associated with DLP were limited to needle costs (assuming that procedure costs would apply to all patients). Needle costs were estimated based on a smoothed average of price data obtained from NHS Wales Shared Services Partnership Procurement Services [14] and are anticipated to be generalisable to UK-wide costing. Resource and costs are reported in Table 2.

**Table 2:** Resource and cost inputs

Model parameter	Unit cost	Source	Description
Needle (atraumatic)	£7.00^a^	NHS Wales Procurement [14]	Approximate cost
Needle (conventional)	£3.00^a^	NHS Wales Procurement [14]	Approximate cost
MM of PDPH	£822.41	NHS Costs, 2020-21 [13]	Weighted average of NEL and NES average costs
EBP for PDPH	£597.77	NHS Costs, 2020-21 [13]	Weighted average of day case average unit costs
No MM of PDPH	£0.00	Assumption	No cost to UK NHS
Resolved PDPH	£0.00	Assumption	No cost to UK NHS

^a^Approximate needle cost based on data obtained from NHS Wales Procurement Services

Abbreviations: EBP, epidural blood patch; MM, medical management; NEL: Non-elective long stay; NES: Non-elective short stay; NHS: National Health Service; UK: United Kingdom

### Model outcomes

Model outcomes comprised patient costs and outcomes based on a hypothetical cohort of 100 DLP patients (broadly typical of 1 week of throughput). Incremental costs were estimated based on the expected costs per PDPH event avoided through use of ATN in preference to CN (all PDPH events and severe PDPH events only). Patients were not explicitly stratified by severity in our primary data source [1] however, based on guideline definitions of treatment, no MM and MM without EBP was assumed to capture mild/moderate PDPH patients, and EBP assumed to capture severe PDPH patients [1]. We also estimated the DLP patient numbers needed to treat (NNT) to avoid one case of PDPH and one case of severe PDPH.

### Treatment of uncertainty

An extensive range of sensitivity analyses (SAs) were conducted where model inputs were systematically changed over a range of plausible values. In SAs 1-and 2, the probability of events was varied within 95% CI limits (relative risk), or by +/-20% (event rate). In SAs 3 and 4 the unit costs of ATN and CN were varied by +/- 20%. In SAs 5 to 7, MM and EBP unit costs were varied. These SAs included greater uncertainty to reflect the fact that there was a deal of uncertainty around expected local costing of the management of PDPH (+/-50%). In addition to these analyses, a final sensitivity analysis, explored the possibility that use of ATN would result in higher failure rates than CN as a proxy for potential ‘learning curve’ effects (+ 20% for the likelihood of a second insertion). Finally, following selected expert feedback suggesting that the underlying rates of DLP-specific PDPH from Nath et al. [1] may be higher than would be expected in practice, we conducted a threshold analysis to see at what rate of underlying PDPH and at what rate of hospital management of PDPH (MM and EBP rates remained proportional) the introduction of ATN would be cost neutral. Probabilistic sensitivity analysis (PSA) was not included in this provisional analysis.

## Results

### Base case outputs

Based on a hypothetical cohort of 100 patients, we estimated 31 cases of PDPH in the CN patients and 12 in the ATN patients, indicating 19 cases of PDPH avoided through use of ATN. Total cohort costs were estimated at £9,469 for CN patients, and £4,257 for ATN patients, a difference of £5,212. This indicates a potential per patient saving of £52.12 through use of ATN in preference to CN.

In the CN arm, of the 31 patients who were predicted to develop PDPH, a total of 12 patients were predicted to need hospital intervention, with 5 requiring readmission/continued hospital stay and 7 needing application of EBP. In the ATN arm, of the 12 patients predicted to develop PDPH, 5 patients were predicted to need hospital intervention, with 2 requiring readmission/continued stay and 3 needing application of EBP. This indicates that for every 100 DLPs conducted using ATN versus CN, we might expect to avoid 3 PDPH-related hospital re-admissions or continued stays and 5 EBPs. The NNT with ATN versus CN in order to avoid one case of PDPH was estimated at 5, with the NNT to avoid one case of hospital managed PDPH (medical management or EBP) estimated at 13.

### Sensitivity analyses and threshold analyses

SA outputs and SA descriptions are reported in Table 3. The impact of plausible variation in inputs on per patient costs ranged between 1% (needle cost) and 54% (upper plausible limit of HCRU costs). When considering a proxy for an ATN learning curve, per patient savings were estimated at £50.39.

**Table 3:** Sensitivity analysis description and outputs

SA #	Parameter	Parameter value			Per patient saving (£)	
		Basecase*	lower	upper	lower	upper
1	p PDPH (CN)	0.31	0.25	0.37	40.80	63.45
2	RR PDPH (ATN)	0.38	0.26	0.55	63.08	36.60
3	CN unit cost	3.00	2.4	3.6	51.45	52.80
4	ATN unit cost	7.00	5.60	8.40	53.70	50.55
5	MM unit cost	822.41	411.21	1233.62	39.69	64.56
6	EBP unit cost	597.77	298.88	896.65	36.25	68.00
7	MM/EBP unit cost	as above	as above	as above	23.81	80.43

*In the basecase, per patient saving was estimated at £52.12

Abbreviations: ATN: atraumatic needle; CN: conventional needle; EBP: epidural blood patch; MM: medical management; p: event probability; RR: relative risk of ATN event compared to CN event; SA: sensitivity analysis

The threshold analyses looked at the point at which the introduction of ATNs would be cost neutral (i.e. no longer cost saving but not involving extra expenditure). We found that when the expected rate of PDPH with CN DLPs reduced below 0.02 (i.e. 2 cases of PDPH (all severity) for every 100 CN DLPs conducted), introduction of ATN would be cost neutral. In this scenario, the difference in cost would be zero however, 2.4 cases of PDPH would still be averted for every 100 patients undergoing DLP. The second threshold analysis found that even if only 3% of PDPH patients were managed in a hospital setting (versus the basecase and literature-supported estimate of 40%), introduction of ATN would be cost neutral compared to use of CNs. Note that in this scenario, use of ATN would still result in avoidance of 19 cases of PDPH per 100 DLPs as the event rates of PDPH were not adjusted.

## Discussion

Our analysis found that a switch to ATN would result in cost savings and substantial improvements in rates of PDPH when compared against use of CNs. Per patient savings in the region of £52 were estimated despite the higher unit cost of ATN compared to CN and, for a cohort of 100 DLP patients, clinical benefits equated to avoidance of 19 cases of PDPH, 8 of which would have required some form of hospital management and 5 of which would have required EBP application. This economic model is the first exploration of the cost-effectiveness of ATN in a UK setting. The model analysis suggests that using ATN is a cost-efficient approach for DLP, with outcomes suggesting a strong economic case for practice switch, alongside an already robust clinical case for switch [1, 2, 6].

The underlying event rates and relative risks used in the model were explored extensively. In the absence of detailed local data, the selected clinical inputs form a solid basis for a provisional exploration of cost-effectiveness but we accept that the data may not mirror all routine practice and that not all data are specific to DLP (for example data on expected medical management rates are taken from a broader cohort, including anaesthetic LP). Presented together, the SA findings indicate that results of the base case analysis are robust, with all scenarios resulting in cost savings. Within this construct, findings were most sensitive to the management cost of PDPH and the baseline estimate of CN rates of PDPH and MM. In these scenarios, per patient savings ranging from £23.81 to £80.43 (management costs of PDPH varied to +/- 50% of base-case values) and from £40.80 to £63.45 (expected rates of PDPH in the CN arm varied between plausible limits), supporting a conclusion that in the case of DLP, the majority cost driver is the rate of PDPH, not the needle cost.

The per patient cost savings found in this analysis are comparable to those presented in the studies identified in our review of the economic literature. The economic review identified four studies relevant to our topic. Two of the studies were conducted in the US [7, 8] one study was conducted in Denmark [10] and one study in the UK [9]. Results from the studies suggested that the use of ATN could lead to a cost saving of between $26 and $142 (broadly equivalent to a GBP range of £20 to £114) per patient when used in place of CN. Our finding of a £52 per patient saving is conservative when compared to the previously published UK study [9], which found a per patient saving of £60 (in 2014 UK£). Differences can be explained by the difference in our approach to the costing of PDPH in that the previous UK study included hospitalisation, GP and A&E visits in their estimate of PDPH cost [9] while, due to the uncertainties around a detailed definition of a PDPH management pathway, we chose to limit our focus to hospitalisation costs. In each study, management of PDPH was the key cost driver, supporting the importance of PDPH avoidance, in line with the findings reported here.

There are a number of limitations to the current study: the clinical evidence base comes from multiple geographies with different practices in LP and limited numbers of DLP-specific studies and baseline rates of PDPH may be higher than those found in standard UK clinical practice (although they are in line with the rates found in the UK study we identified [9]). In addition, our analysis is limited by a lack of detailed costings for the management of PDPH. However, both rates of PDPH and our approach to costing were explored in SA and model findings were robust to their variation. The costing of the PDPH pathway was challenging and findings from the conducted review did not help to clarify a detailed approach or identify cost sources that were specific to PDPH. The analysis settings were diverse and the UK analysis assumptions on costing were not fully referenced [9]. In the absence of a clear approach, we used UK NHS reference cost data [13] as a proxy for expected PDPH costs in the expectation that these costs are of similar magnitude to those reported in the existing literature and are broadly applicable to other settings. This resulted in a similar scale of cost inputs as that seen in other published studies; in general, needle costs were in the tens of dollars/pounds and PDPH management costs (readmission and/or EBP) in the hundreds. Despite this, further UK-based research is needed to better explore UK-specific rates of PDPH and better parametise PDPH costs to increase the robustness of the provisional findings reported here.

We can however use our provisional findings to explore the magnitude of potential cost savings that might be realisable if a practice shift was implemented. Given that our study was conducted under the remit of the NHS Wales, we ran a supplementary analysis that estimated the potential impact of a switch to an increased use of ATNs from the perspective of Welsh LHBs. Research indicates that 4,667 DLPs are conducted on an inpatient (IP) basis in Wales each year (data for outpatient (OP) procedures are not available) [15]. Based on discussion with our clinical collaborators, we assumed that currently only 5% of inpatient DLPs are undertaken with ATNs and that in a single year a plausible change in practice might increase this uptake to 25%. In this scenario, we might expect to avoid 178 PDPH events, 71 of which would be expected to require medical management. Based on the cost assumptions of the model, the switch could result in total cost savings in the region of £48,650, comprising an additional needle spend in the region of £4,200 and a saving through PDPH avoided of £52,800. A crude scaling up to the UK, based on population indices, suggests potential savings in the region of £990,000 if a similar profile of uptake could be expected. This would be in tandem with substantial patient care improvements (over 3,600 PDPH avoided). If outpatient procedures were also included and assumed to be impacted in a similar way, this positive impact would be amplified. Likewise, if the societal costs of PDPH were considered [16].

A previously published editorial cited the in-house availability of ATNs under austerity-led purchasing decisions as a core barrier to ATN uptake [4]. The low uptake of ATN for DLP is reportedly driven by a perception of high costs based on the per unit price of ATN and CN. This perception does not reflect the evidence base for better outcome with ATN and the large cost offsets associated with avoiding even one case of PDPH. These economic arguments have been explored elsewhere but our analysis attempts to quantify them in a UK context. The current study helps raise awareness of evidence-based economic arguments for the use of ATNs in DLP and suggests that a service switch would be both clinically and economically beneficial; marginal differences in needle cost are readily outweighed by a reduction in cases of PDPH and reduced HCRU (a finding which all published data support).

It has been suggested that moving to using ATNs from CNs could require additional training [5]. However, according to the clinical experts we contacted during the development of the economic model, there would be minimal training required and little to no training costs associated with using ATNs. Despite this, further exploration of implementation economics may still be warranted. In a study conducted in France, 176 students underwent specific LP training (including recommendation for ATN use) prior to beginning rotations and were compared to another cohort of students who did not receive specific training for LP [17]. During their clinical examination, 60% of students with specific training used an atraumatic needle compared to 25% in the group of students who did not receive specific training. The authors concluded that specific training in the use of ATN for LP can increase the inclination to use ATNs for LP at a later date and suggest that familiarity with ATNs may be a key driver of ATN use. Inclusion of the cost of these types of initiatives is beyond the scope of our modelling but additional research is warranted in order to ensure behavioural influences of practice change are explored alongside the economic arguments for change.

Our economic model linked available clinical evidence to an expected and locally validated patient pathway. Despite limitations, we constructed a transparent model that draws together a robust clinical evidence base, a locally relevant patient pathway and a provisional attempt at deriving locally relevant costs. The analysis was conducted in full consultation with local experts in NHS Wales and iterative feedback was incorporated and/or addressed in the final study write-up which was submitted to, reviewed and approved by the Evidence Based Procurement Board (EBPB) in February 2020 [18].

## Conclusion

Our analysis found that despite the higher cost of ATNs, a practice shift from CN to ATN is likely to result in substantial savings alongside an overall improvement in patient outcomes through decreased risk of PDPH and its associated management. Prospective data collection, through studies or audits, could further refine this estimation of the impact of a local practice change and increase the robustness of the economic arguments for the positive positioning of ATN in this indication.

## Data Availability

No datasets were generated or analysed during the current study.
